# High-resolution ultrasound of rotator cuff and biceps reflection pulley in non-elite junior tennis players: anatomical study

**DOI:** 10.1186/1471-2474-15-241

**Published:** 2014-07-18

**Authors:** Alberto Tagliafico, Angela Cadoni, Bianca Bignotti, Carlo Martinoli

**Affiliations:** 1 Institute of Anatomy, Department of Experimental Medicine, University of Genoa, Via de Toni 14, 16132 Genoa, Italy; 2 Radiology Department -DISSAL- Università di Genova, Largo Rosanna Benzi 8, 16138 Genova, Italy

**Keywords:** Shoulder, Ultrasound, Tennis, Biceps, Bursitis

## Abstract

**Background:**

Tennis is believed to be potentially harmful for the shoulder, therefore the purpose of this study is to evaluate the anatomy of the rotator cuff and the coraco-humeral ligament (CHL) in a-symptomatic non-elite junior tennis players with high-resolution ultrasound (US).

**Methods:**

From August 2009 to September 2010 n = 90 a-symptomatic non-elite junior tennis players (mean age ± standard deviation: 15 ± 3) and a control group of age- and sex- matched subjects were included. Shoulder assessment with a customized standardized protocol was performed. Body mass index, dominant arm, years of practice, weekly hours of training, racket weight, grip (Eastern, Western and semi-Western), kind of strings were recorded.

**Results:**

Abnormalities were found at ultrasound in 14/90 (15%) players. Two players had supraspinatus tendinosis, two had subacromial impingement and ten had subacromial bursitis. CHL thickness resulted comparable in the dominant and non-dominant arms (11.3 ± 4.4 mm vs. 13 ± 4.2, p > 0.05). Multivariate analysis demonstrated that no association was present among CHL thickness and the variables evaluated. In the control group, abnormalities were found at ultrasound in 6/60 (10%) subjects (sub-acromial bursitis). No statistically significant differences between players and control group were found (p = 0.71).

**Conclusion:**

In a-symptomatic non-elite junior tennis players only minor shoulder abnormalities were found.

## Background

Tennis is practiced by a wide range of people throughout the world and is the most popular of all racket sports. For the last 10 years tennis practice has grown significantly for recreational and competition purposes. Frequently tennis practice begins in childhood and may continue into late adulthood. In spite of the positive effects that tennis practice has shown on physical and mental fitness, some Authors believe that tennis may expose the shoulder to different kind of injuries [1,2]. In non-elite players, efforts spent to develop a more effective and aggressive play using tactics and techniques similar or equal to the elite players are not always supported by an adequate physical training and technical development [1-3]. Shoulder injuries are believed extremely common among elite tennis players and they are not only related to rotator cuff tendinopathy, but also to the long head of the biceps and to the reflection pulley [4-11]; however, the biceps tendinopathy and shoulder dislocations are relatively rare in the young tennis players. It is known that a-symptomatic tennis players may have rotator cuff tendon lesions and reduced sub-acromial space [6-10] and that asymptomatic shoulder abnormalities may be found in the majority of the adults [4]. The rotator cuff interval is the anatomical space where the coracohumaral ligament keeps the long head of the biceps in the appropriate position into the glenohumeral joint. Moreover, when the coracohumeral ligament is intact, the longhead of the biceps does not undergo medial subluxation or dislocation out of the bicipital groove [12]. In case of small anterior supraspinatus tears or shoulder impingement, which may happen in tennis players, the coracohumeral ligament may be thickened and torn. In such cases, the biceps may dislocate over the intact subscapularis and the ruptured lateral part of the coracohumeral ligament can be demonstrated with US [1-14]. For this reasons the integrity of the coracohumeral ligament may be considered as a marker of the integrity of the rotator interval as a whole and as a potential indicator of the technical skills of a tennis players. Indeed, if the technical movements are appropriate, no injuries are expected to occur at the shoulder [15]. Non-elite junior tennis players are supposed to have good technical skills, however in technical learning process some adjustments occur [16]. These adjustments may be responsible of shoulder soft-tissues injuries [17]. To the best of our knowledge, there have been no peer-reviewed ultrasound studies on non-elite junior tennis players including the entire anatomy of the shoulder. It is not known if shoulder abnormalities including rotator cuff tendons and coracohumeral ligament are detectable on high-resolution ultrasound in a-symptomatic junior tennis players.

Therefore, the purpose of our study is to assess if shoulder anatomical abnormalities are present on a complete shoulder ultrasound examination in a-symptomatic non-elite junior tennis players.

## Methods

The study was conducted in accord with the Helsinki Declaration of 1975 and subsequent updates [5]. All enrolled participants provided written consent from a parent or legal guardian. In addition, written consent was obtained from patients or parent/guardian in regards to publication of the patient images. Due to the nature of the study no formal ethical approval was deemed necessary. Tennis players were invited to take part in the study during their practice sessions at their club. To be included in the study, each athlete was required to be a member of the Italian Tennis Federation. To be considered “non-elite” the player had to be an Italian Ranking below 2.8 and not to be involved in National Representatives [18]. The age had to be below 18 years.

For each athlete body mass index, dominant hand (the side on which they held the racquet for the forehand), the number of years playing tennis, the number of hours training per week, and what type of backhand stroke (with one or two hands), racket weight, grip (Eastern, Western and semi-Western), and string stiffness were registered.

**Figure 1:**
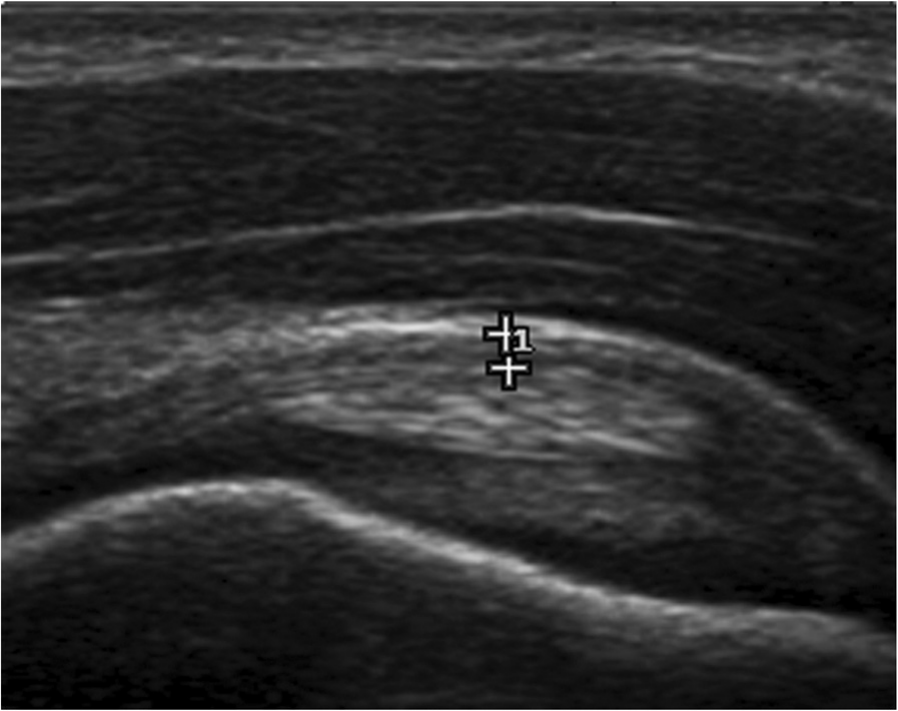
**Example of coracohumeral thickness measurement.** Note the tendon of the long head of the biceps below the calipers.

From August 2009 to September 2010 both shoulder of n = 90 a-symptomatic non-elite junior tennis players (mean age ± standard deviation: 15 ± 3 years) were evaluated bilaterally by mean of high-resolution ultrasound using a 17–5 MHz broadband linear array transducer (iU22, Philips Medical System, the Netherlands). Ultrasonographic evaluation included complete shoulder assessment with a standardized protocol suggested by the European Society of Musculoskeletal Radiology [9] and coracohumaral thickness measurement (Figure 1) as described in literature [19]. All players included in the study had no history of trauma or treatment involving either shoulder. No player had a history of systemic inflammatory disease.

A control group of 60 subjects was constituted by 33 boy and 27 girls (mean age ± standard deviation: 15 ± 3 years). All of them were not involved in overhead recreational or sporting activities and had no history of trauma or treatment involving the shoulder. None of the controls had a history of systemic inflammatory disease. An accurate physical examination was performed before high-resolution ultrasound examination of the shoulder. US scans were performed by two musculoskeletal sonographers (each with more than 5 years of scanning experience): both static images and cine clips were recorded. Recording of static images and cine clips was previously used to analyze US evaluations [20]. The sonographer who performed the scan was blinded to the subject’s dominant side. Both shoulders were scanned. This protocol includes evaluation of the rotator cuff tendons, the tendon of the long head of the biceps brachii muscle in the long and short axes and of the subacromial-subdeltoid bursa, acromioclavicular joint, and posterior recess. Dynamic assessment for subacromial impingement and subluxation and dislocation of the long head of the biceps brachii was also performed. US static images and cine clips were retrospectively reviewed by three musculoskeletal radiologists (3, 4 and 2 years of experience respectively). Only definitive sonographic abnormalities agreed on by the three musculoskeletal radiologists in consensus were included in the study. The ultrasound diagnoses of pathologic findings were based on established criteria and according to the technical guidelines of the European Society of Musculoskeletal Radiology [3,9]. To increase specificity and eliminate false-positive diagnoses, questionable findings were excluded from analysis as suggested by other studies [4].

**Figure 2:**
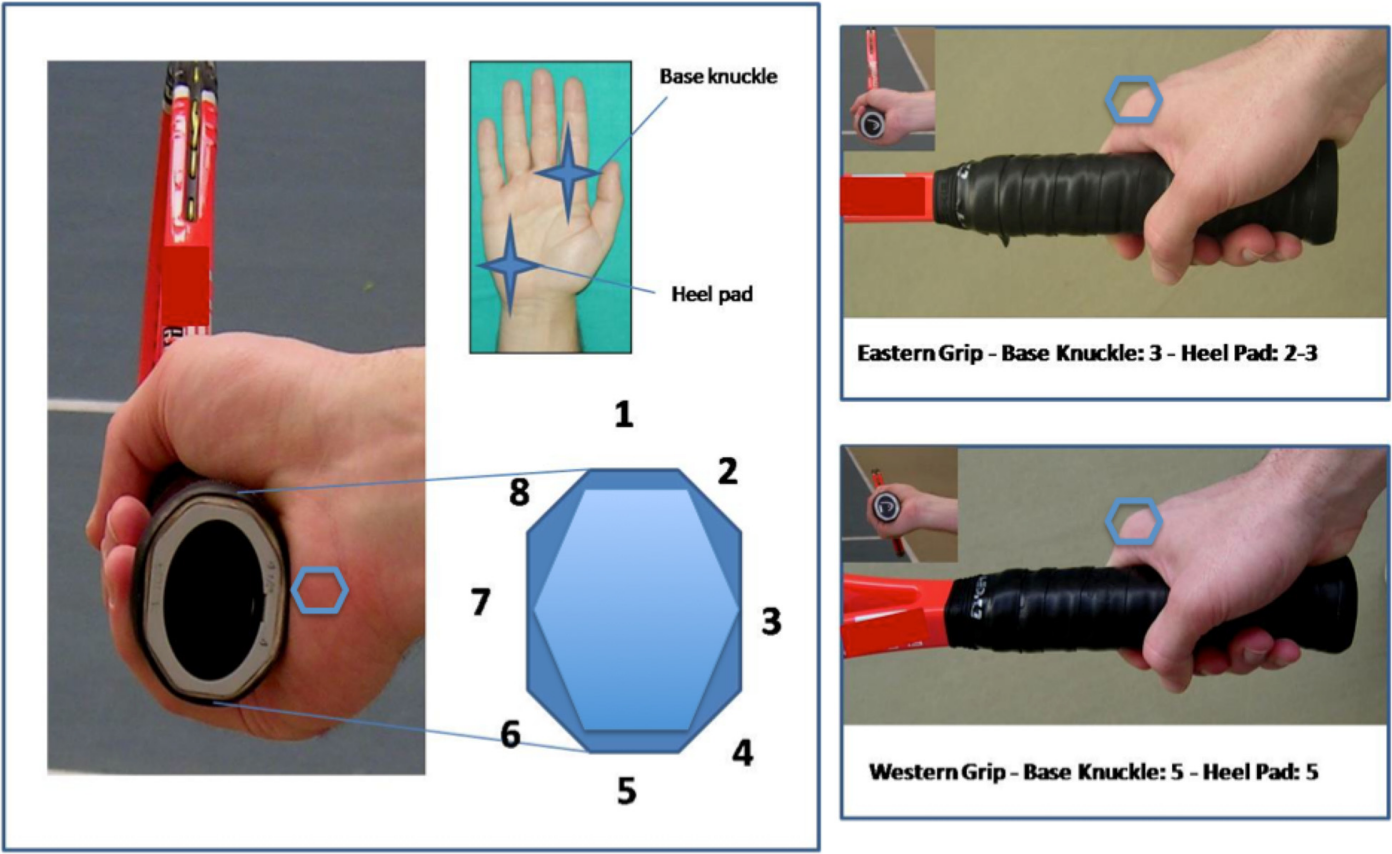
**On the left side are represented the 8 facets of the butt cap and the reference points (base knuckle of the index finger and heel pad) on the hand to identify the different grips.** On the right side the Eastern and Western Grips are illustrated: note that the hand of the players is in the same position while the inclination of the racket changes. Other grips are described in the text. The blue hexagons are positioned in critical areas (base knuckle and heel pad).

Concerning the grip we registered the four basic single-handed grips used to hit the forehand: Continental, Eastern, Semi-western and Full Western. For each grip, the player places the base knuckle of the index finger and the heel pad of the palm on the grip bevel of the racquet. Different grips are defined on the base of the location of the base knuckle of the index finger on the eight faces of the racket grip (Figure 2). Grip types were defined according to the International Tennis Federation definitions [1,21] and checked for accuracy by two tennis instructor in consensus who observed the players holding the racket at rest and during the game.

### Continental grip

In the Continental grip the base knuckle is placed on the face number 2 and the heel pad between 1 and 2. This grip was once the universal grip used to hit almost all strokes: forehands, backhands, special shots, volleys and the serve. It originated on the soft, low bouncing clay courts of Europe. Nowadays it is usually employed only for serves and volleys.

### Eastern grip

In the Eastern grip the base knuckle is on face 3 and the heel pad between 2 and 3. This grip arose on the medium-bouncing courts in the Eastern United States. It represents the classic forehand grip. The eastern grip is appropriate for different styles of play, comfortable for beginners, and adaptable for all surfaces. The advantages of the eastern grip are that it is easy for beginners to learn, it is easy to generate power, it is ideal for waist high balls, and you can hit a variety of topspin, under-spin and flat drive. The disadvantage is that it is difficult to powerfully hit very high balls.

### Semi-western grip

The Semi-western forehand grip has the base knuckle and the heel pad on the face 4. Strength and control to the forehand are guaranteed by this grip, moreover beginners feel comfortable since the palm of the hand supports the racquet providing additional stability at contact. Powerful topspin forehands are the strokes facilitated by this grip. Advantage to this grip is that high balls are easy to hit, however low balls are difficult, back-spin is difficult and grip changes are necessary to hit volleys and overheads.

### Western grip

In the Western grip both base knuckle and heel pad are located on face 5. This grip originated on the high-bouncing cement courts of the Western United States. The drawback of this grip is that it closes the racquet face too soon before contact. This is an excellent grip for high balls and topspin but is awkward for low balls and under-spin. It is widely accepted in the popular media that this grip is the most dangerous for the wrist and that a strong wrist and perfect timing are essential to avoid wrist injuries.

#### Statistical analysis

Statistical analysis included descriptive statistic and coracohumeral thickness was assessed to compare left and right side (dominant and non dominant arm). Fisher’s test was used to compare the presence of lesions in the players and in the control group. The presence of associations between the qualitative variables was evaluated using multivariate analysis. The significance level of 0.05 was adopted. The SPSS software package (release 13.0 for Windows, SPSS) was used. A post hoc power analysis was performed to be sure that the sample size was sufficient to make a meaningful statement. An error level or confidence level of 5% and a ß error level or statistical power (1–ß) of 80% was used and considered acceptable for medical purposes. A sample size of 40 enabled confidence within the required confidence ranges.

## Results

**Figure 3:**
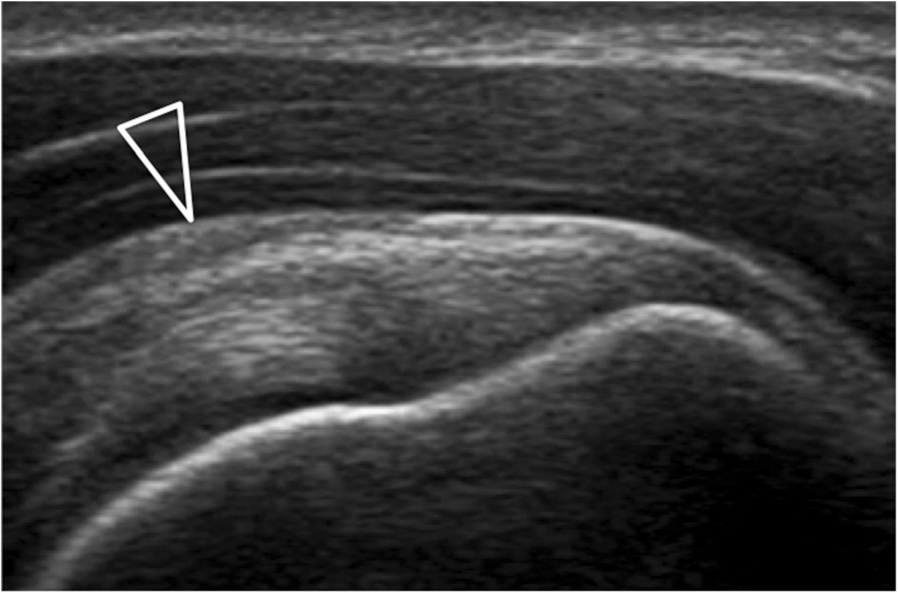
Subacromion subdeltoid bursitis in a 15-year-old tennis player (arrow).

Players’ characteristics relative to sex, dominant arm, years of practice, hours of training per week, grip, racket weight, type of backhand and body mass index are reported in Table 1.Abnormalities were found at ultrasound in 14/90 (15%) players. The majority of the lesions were located in the dominant arm (n = 10), whereas only few of them were in non-dominant arm (n = 4). No tendon tears were detected. Two players had supraspinatus tendinosis. Sub-acromial bursitis was present in 10 players (Figure 3). Two players had subacromial impingement. No rotator-cuff muscular atrophy was found. Coracohumaral thickness resulted comparable in the dominant and non-dominant arm of the players (11.3 ± 4.4 mm in the dominant arm versus 13 ± 4.2 in the non-dominant arm, p > 0.05). Multivariate analysis demonstrated that no association was present among coracohumaral thickness or lesions detected at sonography and body mass index, years of practice, weekly hours of training, racket weight, strings and dominant arm. In the control group, abnormalities were found at ultrasound in 6/60 (10%) subjects (sub-acromial bursitis). No statistically significant differences between players and control group were found (two-tailed P value = 0.71). Coracohumeral thickness was not statistically different in the two groups (p > 0.05).

**Table 1:** Players characteristics

**Number of players**	**M/F**	**Dominant arm right/Left**	**Years of practice**	**Hours of training per week**	**Grip (E/W/SW)**	**Racket weight**	**Backhand stroke (one hand/two hands)**	**Body mass index**
90	46/44	74/16	7 ± 4	3 ± 2	18/36/36	280 ± 32	10/80	22 ± 3

Data are expressed in mean ± standard deviation E, Eastern; SW, semi-Western; W, Western.

## Discussion and conclusions

The main result of this study shows that in a-symptomatic non-elite junior tennis-players only minor shoulder abnormalities are detectable using high-resolution ultrasound.

These abnormalities are not different from those detected in the age- and sex- matched control group. In a previous study, using on high-resolution ultrasound, shoulder abnormalities were found in 96% of asymptomatic subjects [4]. However, in this study the age range was 40–70 years, therefore it is possible that the alteration found were related to normal ageing instead of the daily activity of the subjects. In our study, the players were young and only 15% of them reported a shoulder abnormality. Overall, shoulder abnormalities detected were mild: no partial or full-thickness tears were found. Sub-acromial bursitis was the most frequent find, but no player had fluid in the other recesses or bursae around the shoulder. The absence of rotator cuff muscular atrophy is sufficient to exclude any subclinical irritative or compressive neuropathy. In a paper published on volleyball players it has been shown that the prevalence of infraspinatus muscle atrophy in professional a-symptomatic players is 30% [8]. This data related to volley was not confirmed in our series of young tennis players. We acknowledge that it may be questionable to compare volley to tennis, but both sports are characterized by several overhead strokes that may damage the shoulder. Moreover, the medical literature lacks of similar studies on young non-elite tennis players to be compared with our work. In our series, the majority of the lesions were located in the dominant arm and few of them were located in the non-dominant arm. This observation, although based on few numbers, is not surprising and it may confirm that, in this physical activity, the dominant arm is more stressed than the non-dominant arm. Concerning the grip adopted by the players we did not registered any association between lesions and grips. This observation differs from the fact that, in nonprofessional tennis players, different grips of the racket are related to the anatomical site of the lesion at the wrist: Eastern grip with radial-side injuries and Western or semi-Western with ulnar-side injuries [1]. On the base of this consideration, we can make the hypothesis that the way of hitting the ball with different grips does not influence the biomechanical chain of the stroke at the level of the shoulder. Body mass index and strings stiffness were also considered because they may influence negatively the incidence and pattern of injury [1].

Our study has limitations: the first is the cross-sectional and not prospective design. However, for the purpose of the study, we believe that the design may be considered almost sufficient. Moreover, we did not evaluate an adult population of players, therefore it is difficult to made comparisons with the existing literature and with “veterans” players who received similar loads on the shoulder for several years and hours. In conclusion, the main finding of our study is that shoulder’s soft-tissues of non-elite junior tennis players are similar to age- and sex- matched controls.

## Competing interest

The authors declare that they have no competing interests.

## Authors’ contributions

AT, AC, BB and CM entirely contributed to this paper. All authors read and approved the final manuscript.

## Pre-publication history

The pre-publication history for this paper can be accessed here:

http://www.biomedcentral.com/1471-2474/15/241/prepub
